# Digital psychiatry and COVID-19: the Big Bang effect for the NHS?

**DOI:** 10.1192/bjb.2020.114

**Published:** 2020-10-21

**Authors:** Subodh Dave, Seri Abraham, Roshelle Ramkisson, Shevonne Matheiken, Anilkumar S. Pillai, Hashim Reza, J. S. Bamrah, Derek K. Tracy

**Affiliations:** 1 Derbyshire Healthcare Foundation Trust, Royal Derby Hospital, UK; 2 Pennine Care NHS Foundation Trust, UK; 3 Pennine Care NHS Foundation Trust, UK; 4 Northamptonshire Healthcare Foundation Trust, UK; 5 Bradford District Care Foundation Trust, Horton Park Medical Practice, UK; 6 Oxleas NHS Foundation Trust, UK; 7 Greater Manchester Mental Health Foundation Trust, UK; 8 Oxleas NHS Foundation Trust, UK

**Keywords:** Telepsychiatry, digital psychiatry, COVID-19, education and training, information technologies

## Abstract

The COVID-19 pandemic has brought untold tragedies. However, one outcome has been the dramatically rapid replacement of face-to-face consultations and other meetings, including clinical multidisciplinary team meetings, with telephone calls or videoconferencing. By and large this form of remote consultation has received a warm welcome from both patients and clinicians. To date, human, technological and institutional barriers may have held back the integration of such approaches in routine clinical practice, particularly in the UK. As we move into the post-pandemic phase, it is vital that academic, educational and clinical leadership builds on this positive legacy of the COVID crisis. Telepsychiatry may be but one component of ‘digital psychiatry’ but its seismic evolution in the pandemic offers a possible opportunity to embrace and develop ‘digital psychiatry’ as a whole.

The COVID-19 outbreak has posed an unprecedented challenge to healthcare systems and to society as a whole, with millions infected globally and tens of thousands of deaths in the UK alone. Stay-at-home and lockdown guidance was instituted to ensure that the National Health Service (NHS) had the capacity to deal with a possible surge in COVID-19-related presentations, not least as NHS staff were equally affected by quarantine and sickness challenges. Remote consultations, or ‘telemedicine’, hitherto a somewhat niche offering in the NHS led primarily by technology enthusiasts, saw an explosive growth, with the New York Times reporting: ‘Telemedicine arrives in the U.K.: “10 years of change in one week”’.^1^

The urgent need to deliver patient care effectively and safely in the pandemic was supported by new NHS guidance^2^ that facilitated a rapid rollout of remote consultations in most areas of psychiatric practice.

This occurred despite the pre-existing cautionary digital guidance from the Royal College of Psychiatrists echoing the General Medical Council (GMC) in its advice that ‘standards expected of doctors […] apply equally to digital and conventional consultation settings’ and that doctors must give ‘consideration to the potential limitations of the medium used’.^3^ In particular, for psychiatrists, perhaps more than any medical specialties, there have been long-standing concerns of loss, potentially harmfully so, of the ability to pick up by distance the subtle interpersonal cues of the mental state examination.

‘Telepsychiatry’ is not new. Defined as the provision of psychiatric services remotely through various technological communication platforms,^4^ technology-based mental healthcare has been in operation for several decades. Samaritans, the well-known telephone helpline service, started its operations in 1953. However, a more important contemporary phrase is ‘digital psychiatry’, which moves beyond just the digital delivery of a consultation to the provision of an on-demand, highly personalised, confidential and secure care made available through an easy-to-use, intuitive interface. However, digital psychiatry is much more than that. Rethinking conventional psychiatry, heavily reliant on the written and printed word to store medical information, often distributed by traditional methods such as by post, to move to a world where digital tools can record, analyse and make intelligent interpretations of data will require a clinical, administrative and intellectual evolution.

The NHS Long Term Plan outlines its vision of every patient (in England) being able to access digital services – at least at the primary care level – by 2024, putatively saving 30 million patient trips and over £1 billion/year in costs.^5^ However, the possibility of delivering a socially distanced but safe and effective service has provided a fresh fillip for the adoption of digital health in both primary and secondary healthcare. Will the relatively enthusiastic adoption of virtual assessments provide a template for the wider rollout of new technologies in clinical practice beyond the pandemic?

If such promise is to be realised, remote consultations, digital self-help, electronic patient record systems, triaging using artificial intelligence (AI) and a range of other digital tools will need to be scaled up sustainably, paying attention to patient preference, patient safety and clinical outcomes, including the concept of precision psychiatry. This article outlines the key factors that need urgent consideration to ensure the integration of digital solutions in routine clinical practice.

## Is digital psychiatry safe, effective and acceptable to patients and clinicians?

The justifiable clinical concern of coronavirus infection during the pandemic has shifted the fulcrum of safety significantly away from face-to-face consultations. The use of telemedicine in post-disaster situations is well-established.^6^

Systematic reviews demonstrate substantial evidence to support the effectiveness of remote psychiatry.^7,8^ Most of the evidence has emerged from the need to overcome accessibility barriers to psychiatric care, for example in areas isolated owing to disasters such as hurricane Katrina or to their geographical location, such as rural Canada and Australia. Studies looking at the effectiveness of remote clinical work are mainly head-to-head and non-inferiority studies demonstrating equivalence with face-to-face interactions. These show effectiveness comparable with face-to-face assessments in terms of patient engagement, validity/reliability of assessments, and clinical outcomes.^9,10^

Rapidly emerging evidence shows that telepsychiatry is being successfully utilised globally, in the wake of the COVID-19 pandemic. Yellowlees et al^11^ reported the process, challenges and lessons learned from a rapid conversion of a direct psychiatric clinic to a virtual one within the space of 3 working days in Northern California. Rosic et al^12^ provided an interesting patient and provider perspective on the transition to a virtual clinic following the onset of the pandemic in Canada. Sharma et al^13^ have discussed a similar transition in a child and adolescent setting in Seattle. Duan & Zhu^14^ have described the development of mobile phone and social media-based platforms to provide psychological care in China. These examples demonstrate the point that COVID-19 has propelled the global psychiatric community into a new era with the use of technology in delivery of psychiatric care.

Remote access has been noted to be effective for treating a variety of conditions, such as depression, anxiety, post-traumatic stress disorder (PTSD) and psychosis. It has been particularly useful for individuals who face specific difficulties attending out-patient appointments, such as some with psychosis and social anxieties, and social difficulties such as housing instability.

In child and adolescent psychiatry, we have evidence for the effectiveness of digital psychiatry for the psychiatric assessment and management of attention-deficit hyperactivity disorder and early psychosis, as well as delivery of therapy for obsessive–compulsive disorder and tic disorder.^15^ Hantke et al^16^ demonstrated the effectiveness of remote working in a variety of older person's settings, such as nursing home, community and hospital settings for individuals with cognitive, functional and sensory impairment. Such interventions have also been associated with reduced transfers to hospital and benefits for patients with limited mobility.

Benefits have been shown in criminal justice and other forensic settings, where safety concerns about physical movement of high-risk patients can be mitigated through remote assessments and remote expert testimony.^17^ Remote psychiatry has also been demonstrated to have a positive impact on the assessment and management of individuals with intellectual disabilities, with some data showing no loss in therapeutic engagement compared with face-to-face assessments and even some evidence for improved engagement among children with severe anxiety and autism.^18^ In addiction psychiatry, remote treatment of opioid use disorder produced similar outcomes as face-to-face treatment in both general^19^ and obstetric settings.^20^

Interestingly, healthcare providers were more likely than patients to express concerns regarding adverse effects of remote assessments on therapeutic alliance.^7^ Although there is considerable evidence for remote therapy,^21^ Norwood et al^22^ found that working alliances were inferior compared with face-to-face work, even though symptom reduction was equivalent.

However, the (few) studies on the topic tend to report overall high levels of satisfaction with digital consultations,^23^ including for children and adolescents.^15,24^

Bashshur et al^25^ identified telemedicine as a cost-effective solution for triage, consultation, prescribing medications, provider-to-provider discussions, appointment scheduling and reminders. Furthermore, the study also found that remote interventions in primary care were at least as effective as traditional care.

Given these effectiveness and experience data and the evidence that telepsychiatry is cost-effective compared with face-to-face treatment, one must question the historical factors limiting adopting technological solutions in the value-driven public NHS.

## Potential barriers to implementing digital psychiatry

The potential barriers to digital working fall into three broad categories – regulatory concerns, technological hurdles and human factors.

NHS practice is influenced by several bodies, including the GMC, the medical Royal Colleges and medical defence unions, clinical guidance from the National Institute for Health and Care Excellence (NICE), and local and national commissioning protocols involving a host of stakeholders, such as clinical commissioning groups, NHS trusts and so forth. None preclude digital working, and indeed all generally support the principles. However, their nature, number and potentially conflicting messaging can make them inherently resistant to swift changes, even for interventions with proven clinical benefit and cost-effectiveness.

There is a wide variation in the technological maturities among mental health providers across the country. Technological and security concerns include clinical governance issues, safeguarding, legal liability, confidentiality and secure storage of digital information, with worries about reliability of technology and variation in bandwidth across the country.

Human factors can be a potent barrier, with clinician anxieties centring on: building rapport in a digital interview; being ‘recorded’, with potential consequences for personal liability; and perhaps most powerfully, the lack of personal incentives to change. The densely populated nature of the UK may make some accessibility problems seem less relevant. The edict of *primum non nocere* – first do no harm – is so embedded in medics’ psyche that it perhaps makes clinicians inherently cautious about change. This is likely only exacerbated by many doctors’ adverse perceptions of restrictive, stifling information governance rules and regulations.

For patients and carers, lack of access to technology on account of financial, technological, physical or cognitive factors may be a barrier. Global evidence, however, demonstrates that patients show a clear preference to having alternatives in addition to face-to-face assessments.

The COVID-19 crisis demonstrated that all these barriers can be rapidly overcome. NHSX provided timely and much needed guidance and assurance on the use of a variety of methods to enable and support working.^3^ Despite inevitable hiccups, internet and technology solutions worked in a manner perhaps not attainable had the pandemic occurred say even 5 years ago. Clinician and patient experiences have surely buried the ‘unacceptable’ argument.

## Digital psychiatry: the future

Recent experiences have exemplified the differences between older-fashioned ‘telepsychiatry’ and the innovations possible with ‘digital psychiatry’. Doing ‘the same’ but via video calls is limited progress, though perhaps the initial leap made by most clinicians. A variety of platforms have been made available with new features such as: a waiting room (simulating clinics); multiple participants (to enable multidisciplinary team working); a screen sharing feature to show written information (to aid explanations and education during the clinical interview); and inbuilt capability to email or message the patient, carer or other colleagues and to save these communications directly in the electronic clinical records. Recent developments have also shown clinician benefits beyond ‘just’ the flexibility of working from home, including examples of offering more flexibility in hours of work and timings of clinics, such as evening and weekend working (no longer needing office buildings to be kept open). Asynchronous meetings are allowing staff to read, comment on and contribute to documents outside of the ‘standard meeting’ time, as well as message and add written comments as meetings progress.

Simultaneously, there have been some anecdotal concerns about ‘sharing’ one's home environment with others, whether colleagues or patients, alongside some sense of fatigue at engaging many participants online without full human engagement. The range of competing platforms is potentially confusing and we are still learning to navigate these, and when and how to use the novel technologies within.

But while we attempt to master the etiquette of how and when to speak across large meetings, digital solutions have also emerged to support home-based care for our patients. From the surge in the use of digital apps for mental health and well-being to the rise in digital prescribing, digital dictation and even digital therapies as people adapted to the lockdown world, technology has felt more palpable for both clinicians and patients. Innovations such as AVATAR therapy for auditory hallucinations in chronic schizophrenia or individualised risk stratification using AI machine learning to ‘read’ patient records that are currently being piloted seem that much closer to routine practice.

Although the outcomes for patients receiving digital psychiatric care do not seem to be inferior to in-person care, we need better data about the subgroups of patients for whom this might not hold true. For example, early evidence suggests caution for individuals with cognitive impairment, at high-risk, with significant concomitant physical health needs and so forth. National data-sets from agencies such as NHS Digital or the Care Quality Commission may help inform this.

The legal, ethical and regulatory framework relating to remote consultations also needs clarification. Both patients and clinicians need to feel safe participating, and key issues include consent, capacity, confidentiality, need for chaperones, safeguarding for vulnerable patients, escalation arrangements, security of data and indemnity for clinicians. Early and successful resolution of these issues will avoid the stifling of innovation and will enable a more rapid adoption of wider digital enhancers to patient care.

The theme of integration is key to the NHS Long Term Plan and features prominently in the new community mental health framework.^26^ Digital psychiatry in its broadest sense offers a unique opportunity to realise this integration, albeit virtually, of primary care, social care, third-sector partners, the criminal justice system and other stakeholders working with patients and clinicians to improve clinical outcomes. From virtual meetings to seamless patient-owned records, the possibilities are limitless.

A central feature of UK health policy and service delivery is its focus on person-centred care and this is particularly true for psychiatric practice and training.^2^ Co-production has largely been absent in the evolution of digital psychiatry and it is vital that patients and carers have a central role in further rollout of this new technology. Current guidance issued for the pandemic will need to be updated on the basis of emerging evidence on indications and pathways and it will also need to take into account patient and professional feedback.

Workforce implications will need to be carefully considered. Roles and responsibilities for clinicians working remotely need to be clearly defined. Current arrangements enable the enforcement of national regulatory jurisdictions, and this might be challenged by some forms of virtual working. For example, licensure arrangements across several states in the USA have been relaxed to allow licensed clinicians to work remotely from outside normal state boundaries. This may be particularly relevant for countries such as the UK, where there has been a traditional reliance on international healthcare workers to provide an adequate clinical service: both an opportunity and challenge in working with clinicians outside of traditional workforce bases emerge.

Finally, the workforce will need appropriate training to deliver remote consultations safely and effectively. Currently, in the UK there are no curricula-specific training requirements, either at core or higher specialty level, for psychiatry trainees to demonstrate competence in digital skills that may be considered essential to good clinical practice, e.g. managing digitally enabled consultations, extracting clinically meaningful data from electronic patient records or prescribing evidence-based digital apps. Examinations may be moving online, as is the case with MRCPsych examinations beginning later this year, but embedding digital literacy in the training and assessment framework will require a significant shift in culture and practice.

## Conclusions

The COVID-19 pandemic has given the NHS permission to rapidly review its ways of working to embrace technological advances. These offer the potential of flexible home-based consultations for clinicians and patients; the opportunity to connect multiple agencies more quickly to deliver a person-centred care plan; accessibility to communities who might otherwise not be reached; a window into the personal and home life of our patients; and all this potentially with a smaller carbon footprint and lower costs.

However, if we are to fully tap into the potential gains of digital psychiatry, we must realise how much more than this is on offer: an integrated use of technology in mental healthcare, supported by multidisciplinary, diverse teams of technologists, designers, health and care professionals and those with lived experience. It is about agile methodologies, user research, behaviour-change science, data science and social science blending together in organisations with less hierarchical power play and a more pragmatic and courageous approach to risk, as has been the case during this pandemic.

Our aspiration for digital psychiatry should reflect the expectations of the internet age – on-demand entertainment on a mobile digital device, real-time customer logistics so that one knows where a parcel is and the name of the driver, universal standardisation of our experience through ‘operating systems’ that allow fine-grained personalisation. We have much more to achieve than remote consultations, and certainly far more than doing video calls. And it is truly ‘digital’ platforms – ubiquitous computing through standardised operating frameworks on highly personalised and network-connected mobile devices – that have allowed us to achieve the adoptions we have in a matter of months.

We propose that what we have described as the barriers to adopting digital psychiatry are solved through harnessing the values, culture, practice and technological capabilities of the internet age.

Although the growth of digital psychiatry in the NHS may have been more of an evolution than a revolution, with the right leadership, training, research on digital innovations, and the necessary clinical, ethical and legal guidance we can dispel the digital darkness and usher in a new era of integrated, personalised and accessible psychiatric care. We call on the Royal College of Psychiatrists to set up a task force to develop national guidance to ensure that the Big Bang effect of COVID-19 on digitisation of clinical practice and training is sustained and amplified in the future.

## About the authors

**Subodh Dave**, FRCPsych, is a consultant psychiatrist with Derbyshire Healthcare Foundation Trust, working at the Radbourne Unit, Royal Derby Hospital, Derby, UK. **Seri Abraham**, MBBS, MRCPsych, MSc, is a consultant psychiatrist with Pennine Care NHS Foundation Trust in Oldham, Greater Manchester, UK. **Roshelle Ramkisson**, MBBS, MRCPsych, PGDip, MSc, is a consultant psychiatrist and Director of Medical Education, Pennine Care NHS Foundation Trust, Oldham, Greater Manchester, and Training Programme Director for Core Psychiatry in the North West School of Psychiatry. She also holds a NICE Fellowship. **Shevonne Matheiken**, MBBS, MRCPsych, is a Speciality Doctor at the Northamptonshire Healthcare Foundation Trust and an education attaché at the Pennine Care NHS Foundation Trust, UK. **Anilkumar S. Pillai**, MRCPsych, is a consultant old age psychiatrist with Bradford District Care Foundation Trust, working at Horton Park Medical Practice, Bradford, and Training Programme Director in Old Age Psychiatry with Health Education England Yorkshire and Humber, UK. **Hashim Reza**, MBBS, MRCPsych, is a specialist advisor in health informatics at the Royal College of Psychiatrists, and a consultant psychiatrist with Oxleas NHS Foundation Trust, London, UK. **J. S. Bamrah**, FRCPsych, is a consultant psychiatrist with Greater Manchester Mental Health NHS Foundation Trust, UK. **Derek K. Tracy**, FRCPsych, is a consultant psychiatrist and Clinical Director at Oxleas NHS Foundation Trust, London, and a senior lecturer at the Institute of Psychiatry, Psychology and Neuroscience, King's College London, UK.

## Supplementary material

For supplementary material accompanying this paper visit https://doi.org/10.1192/bjb.2020.114. click here to view supplementary material
